# Interleukin 6 (rs1800795) and pentraxin 3 (rs2305619) polymorphisms-association with inflammation and all-cause mortality in end-stage-renal disease patients on dialysis

**DOI:** 10.1038/s41598-021-94075-x

**Published:** 2021-07-20

**Authors:** Susana Rocha, Maria João Valente, Susana Coimbra, Cristina Catarino, Petronila Rocha-Pereira, José Gerardo Oliveira, José Madureira, João Carlos Fernandes, Maria do Sameiro-Faria, Vasco Miranda, Luís Belo, Alice Santos-Silva, Elsa Bronze-da-Rocha

**Affiliations:** 1 LAQV, REQUIMTE, Laboratório de Química Aplicada, Departamento de Ciências Químicas, Faculdade de Farmácia, Universidade do Porto, Porto, Portugal; 2 UCIBIO, REQUIMTE, Laboratório de Bioquímica, Departamento de Ciências Biológicas, Faculdade de Farmácia, Universidade do Porto, Porto, Portugal; 3 CESPU, IINFACTS, Gandra, Paredes, Portugal; 4 Centro de Investigação em Ciências da Saúde, Universidade da Beira Interior, Covilhã, Portugal; 5 Centro de Investigação em Tecnologias de Saúde (CINTESIS), Faculdade de Medicina, Universidade do Porto, Porto, Portugal; 6 Clínica de Hemodiálise do Porto, Porto, Portugal; 7 Centro de Hemodiálise de Nossa Senhora da Franqueira, NefroServe, Barcelos, Portugal; 8 Centro de Hemodiálise de Viana do Castelo, NefroServe, Viana do Castelo, Portugal; 9 Unidade de Hemodiálise, Hospital Agostinho Ribeiro, Felgueiras, Portugal; 10 Clínica de Hemodiálise de Gondomar, Gondomar, Portugal

**Keywords:** Kidney, Kidney diseases

## Abstract

Chronic inflammation plays an important role in the progression and outcome of chronic kidney disease (CKD). The circulating levels of the inflammatory biomarkers interleukin 6 (IL6) and pentraxin 3 (PTX3) are enhanced in CKD patients, and are associated with the progression of the disease and with higher risk for cardiovascular events, the major cause of death in CKD patients. Our aim was to study how specific polymorphisms of IL6 and PTX3 encoding genes affect the inflammatory response and outcome of end-stage renal disease (ESRD) patients on dialysis. Methodology included the analysis of two single nucleotide polymorphisms (SNP), namely the *IL6* (rs1800795) polymorphism in the promoter region (-174G > C), and the *PTX3* (rs2305619) polymorphism in the intron 1 (+ 281A > G), which were analyzed in ESRD patients on dialysis and in a group of heathy individuals. The allelic frequencies, genotype distribution and their association with circulating levels of the inflammatory markers C-reactive protein (CRP), IL6, growth differentiation factor 15 (GDF15) and PTX3, were determined in ESRD patients. Events of death were recorded along one year, to assess the association of the studied SNPs with all-cause mortality and the inflammatory biomarkers, in ESRD patients. Results showed that the allelic frequencies and genotype distribution for *IL6* and *PTX3* SNPs in the control group and ESRD patients were similar and in agreement with other European reports. For the *IL6* polymorphism, we found a trend towards higher levels of high-sensitivity (hs) CRP, IL6 and PTX3 in the homozygous genotypes; the CC genotype also showed the highest levels of GDF15. The mortality rate after the 1-year follow-up was 10.4%. The CC genotype (*IL6* SNP) was associated to a higher risk of mortality and deceased patients carrying this genotype also showed the highest levels of hsCRP. Regarding the studied *PTX3* SNP, the AA genotype was linked to an enhanced inflammatory response, showing the highest values of hsCRP and IL6. Nevertheless, this genotype had no significant impact on the mortality rate. In conclusion, both studied SNPs seem to modulate the inflammatory response in ESRD and may, therefore, be determinant on disease progression and patients’ outcome. Our data also highlights the importance of research on genetic variants that, although less frequent, may have significant biological value.

## Introduction

Chronic kidney disease (CKD) is characterized by a progressive loss of renal function, measured by a decline in the estimated glomerular filtration rate (eGFR), that aggravates from stage 1 to the terminal stage 5 [end-stage renal disease (ESRD)], which requires kidney replacement therapy (KRT) through transplantation or dialysis^1–3^. By 2010, the number of ESRD patients undergoing KRT worldwide exceeded 2.6 million (78% on dialysis and 22% with a kidney transplant), and this number was projected to rise up to 5.4 million by 2030^4^. CKD represents, thus, a major public health problem worldwide.

Inflammation is a common feature in CKD patients, which commonly increases with the severity of the disease. C-reactive protein (CRP), growth differentiation factor 15 (GDF15), pentraxin 3 (PTX3) and interleukin (IL6) are inflammatory mediators whose circulating levels are increased in CKD patients, and particularly enhanced in patients on hemodialysis (HD)^5–7^. The accumulation of uremic toxins, fluid overload, the development of oxidative stress, among other CKD disturbances, contribute to the rise in IL6; the decreased renal function, by reducing IL6 clearance, also contributes to the increase in its levels. Likewise, the dialysis procedures contribute to stimulate the inflammatory response, further increasing IL6 production. The continuous high levels of inflammatory cytokines, have been associated with a high risk for cardiovascular diseases (CVD), which are the major cause of death in these patients^5,6,8–11^.

There are several genes correlated with biochemical and inflammatory markers in CKD, and their genetic variants were associated with kidney (dys)function and with the prevalence of CKD^1,12,13^. However, the relationship between the genetic polymorphisms of some inflammatory markers and the outcome of CKD patients is far from clarified.

The human *IL6* gene is located on chromosome 7p21, has 5 exons and 4 introns and several polymorphisms in the promoter region [*IL6* (-174G > C): rs1800795,  *IL6* (-572G > C): rs1800796, *IL6* (-597G > A): rs1800797, and *IL6* (-634C > G)]^14^. Different studies in ESRD patients on dialysis treatment reported the contribution of *IL6* allelic variants in the promotor region to the variation/modulation of several biochemical and clinical parameters, such as serum erythropoietin, hemoglobin concentration^15^, inflammatory biomarkers^16^, dysfunction in the vascular access for HD procedure^17^, progression of kidney dysfunction^18^, and in the risk for CVD, coronary artery disease^19,20^, and atherosclerosis^21^. However, the influence of *IL6* genetic variants in the inflammatory response, outcome and mortality risk in ESRD still requires more research.

The human *PTX3* gene, located on the long arm of chromosome 3 (q24-28), presents a 5’-UTR and 3’-UTR regions, two introns and three exons^22–24^. The *PTX3* polymorphism (+ 281A > G) (rs2305619) is located at intron 1 (boundary)^25^. Some reports state the association of this polymorphism with susceptibility to diabetic nephropathy^26,27^ and also its association with PTX3 plasma levels and cardiovascular disease events^25,28^. *PTX3* genetic variants [*PTX3* (+ 1449 A > G), second intron: rs1840680, *PTX3* (+ 734A > C), second exon: rs3816527, and *PTX3* (C > A) 5’-UTR: rs2120243] have been associated with several clinical conditions, such as infections, female fertility, risk and progression of oral cancer^29^, type II diabetes nephropathy^27^, hypertension^30^ and migraine^31^. The circulating levels of PTX3 are elevated in CKD patients and appear to independently predict cardiovascular complications^5^. Still, few studies investigated the association of *PTX3* polymorphisms with inflammatory markers of ESRD.

The purposes of this study were to: (1) determine the allelic frequencies and genotype distribution of the genetic variants of *IL6* (rs1800795) and of *PTX3* (rs2305619) SNPs in a Portuguese cohort of ESRD patients on dialysis and in healthy individuals (controls); (2) identify the association of both SNPs with key biomarkers of the inflammatory response, namely, IL6, CRP, GDF15 and PTX3; and (3) assess the influence of these SNPs on all-cause mortality after a 1-year follow-up study with the ESRD cohort.

## Material and methods

### Subjects

The studies and data analysis involving human samples were approved by the Ethics Committee from the Faculty of Pharmacy, University of Porto (Report No. 26-04-2016) and by the National Data Protection Commission (Proc. No. 762/ 2017; Authorization No. 532/ 2017). Blood samples were collected after informed consent of all participants. The inclusion and exclusion criteria for healthy individuals (Control group) and ESRD patients on dialysis treatment, are described in Table 1.

**Table 1:** Inclusion and exclusion criteria for controls and end-stage renal disease (ESRD) patients on hemodialysis (HD) treatment.

	Control	ESRD on HD
**Enrolment criteria**		
Age, years	> 18	> 18
Time on dialysis treatment	–	> 3 months
Enrolled participants, *n*	44	308
**Excluded participants, *n***		
High cholesterol	1	–
Arterial hypertension	6	–
Antiphospholipid syndrome	1	–
Prediabetes	1	–
Under chronic drug treatment	3	–
Active infection	–	14
Neoplasia	–	5
Included participants, *n*	32	289
**Participants excluded during the follow-up period, *n***		
Kidney transplant	–	14
Renal function recovery		2
Clinic transfer		3
Dialysis abandonment		2
Participants included in the 1-year follow-up study, *n*	–	268

Out of 44 enrolled volunteers, the final control group included 32 healthy subjects, with normal hematological and biochemical data, and no history of renal disease. Clinical data from 308 ESRD patients were collected at the Dialysis Clinics in the beginning of the study. 19 patients were excluded from the study due to pre-existent active infections or neoplasia (Table 1). Along the following year, 289 patients were followed, to identify cases of death; a total of 28 deceased patients was reported over the one-year follow-up period, with miscellaneous causes of death, including cardiovascular causes, cachexia, infectious diseases, or others. During this follow-up period, 21 patients left the study for various motives (Table 1). Demographic, clinical and inflammatory data, for controls and ESRD patients on HD treatment, are presented in Table 2.

**Table 2:** Demographic, clinical and inflammatory data for Controls (*n* = 32) and ESRD patients on hemodialysis treatment (*n* = 289).

	Control	ESRD	*p* value
**Gender, *n* (%)**			
Male	13 (40.6)	158 (54.7)	*0.131*
Female	19 (59.4)	131 (45.3)	
Age, years	55.8 ± 4.8	68.7 ± 13.6	***< 0.001***
**Etiology of CKD, *n* (%)**			
Diabetic nephropathy	–	101 (34.9)	–
Hypertensive nephrosclerosis		36 (12.5)	
Polycystic kidney disease		19 (6.6)	
Chronic glomerulonephritis		23 (8.0)	
Other		44 (15.2)	
Undetermined		66 (22.8)	
**Dialysis** **vintage,** **years**	–	3.74 (1.65–7.34)	–
**Dialysis therapy, *n* (%)**			
Hemodialysis	–	41 (14.2)	–
On-line hemodiafiltration		248 (85.8)	
**Vascular access, *n* (%)**			
Arteriovenous fistula	–	233 (80.6)	–
Arteriovenous graft		14 (4.8)	
Catheter		42 (14.5)	
**Inflammatory biomarkers**			
Leukocytes × 10^9^/L	5.30 (4.55–6.50)	6.31 (5.29–7.62)	***0.004***
hsCRP, mg/dl	0.15 (0.04–0.26)	0.37 (0.16–0.81)	***< 0.001***
IL6, pg/ml	1.12 (0.74–1.62)	4.10 (2.69–7.33)	***< 0.001***
PTX3, ng/ml	0.58 (0.42–0.73)	1.40 (0.98–2.05)	***< 0.001***
GDF15, ng/ml	0.96 (0.80–1.09)	10.77 (7.90–13.74)	***< 0.001***

Data are presented as mean ± standard deviation or as median (interquartile range). Multiple comparisons between groups were performed by the Pearson’s Chi-squared test, by the one-way Anova with Bonferroni post-hoc tests or by the Mann–Whitney U test, as appropriate. Italic denotes *p-*values and bold-italic statistically significant *p-*values.

*CKD* chronic kidney disease, *GDF15* growth differentiation factor 15, *hsCRP* high-sensitivity C-reactive protein, *IL6* interleukin 6, *PTX3* pentraxin 3.

### Samples

Blood samples, from both controls and patients, were collected into tubes, without and with anticoagulant (K3-EDTA), and processed within 2 h to obtain serum, plasma and buffy-coat, respectively. Aliquots of the samples were immediately stored at − 80 °C until assayed. Sample collection from ESRD patients took place immediately before a midweek dialysis therapy session.

### Assays

Total leukocyte cell count was assessed in whole-blood using an automatic blood cell counter (Sysmex K1000; Sysmex, Hamburg, Germany).

All inflammatory biomarkers were analyzed through commercially available kits, according to the manufacturer’s instructions. Plasma samples were used to quantify PTX3 (Human Pentraxin 3/TSG-14 Quantikine ELISA Kit, R&D Systems, Minnesota, USA; sensitivity 0.026 ng/mL) and GDF15 (Human GDF15 ELISA Kit, Abcam, Cambridge, UK; sensitivity 2 pg/mL) through enzyme-linked immunosorbent assays (ELISA). In serum we measured IL6 by ELISA (Human IL6 Quantikine HS, R&D Systems; sensitivity 0.09 pg/mL) and hsCRP by immunoturbidimetry [Cardiac C-Reactive Protein (Latex) High Sensitive assay, Roche Diagnostics, Basel, Switzerland; sensitivity 0.15 mg/L].

Genomic DNA was extracted from buffy-coat samples, using genomic DNA extraction kit (GRiSP, Research Solutions, Porto, Portugal), quantified by NanoDrop-1000 (ThermoFisher Scientific, Wilmington, DE, USA) and analyzed by agarose gel electrophoresis. Trademark TaqMan SNP genotyping assays (Human; ThermoFisher Scientific) were performed to assess the allelic frequencies of *IL6* (rs1800795) and *PTX3* (rs2305619) polymorphisms, using a real-Time PCR system (StepOnePlus, ThermoFisher Scientific).

### Statistical analysis

Data were analyzed using the IBM SPSS software, version 25 for Windows 10 (IBM, New York, USA). Data distribution was evaluated by the Shapiro–Wilk test. Results are presented as median (interquartile range), since most variables presented a non-Gaussian distribution. For categorical variables, the comparison between groups at baseline was analyzed using the Chi-squared test. For continuous variables, differences between groups were evaluated using Mann–Whitney *U* test. The strength of the correlations between variables was determined through the Spearman’s rank correlation coefficient. Survival distribution comparisons between genotypes was performed by the log-rank test. Estimation of all-cause mortality hazard ratio (HR), according to the *IL6* polymorphic genotype, was determined by multiple Cox regression analysis. A *p* < 0.05 value was considered statistically significant.

## Results

### Genotype prevalence and allelic frequencies of *IL6* and *PTX3* polymorphisms in ESRD patients and controls

Genotype prevalence and allelic frequencies in ESRD patients and controls were analyzed for both SNPs of *IL6* rs1800795 (-174G > C) and of *PTX3* rs2305619 (+ 281A > G) (Table 3).

**Table 3:** Genotype and allelic distribution of *IL6* (-174G > C) and *PTX3* (+ 281A > G) polymorphisms in controls (*n* = 32) and end-stage renal disease patients on dialysis (*n* = 289).

*IL6* (-174G > C)	Control	ESRD	*p* (*χ*^2^)
Genotype	*n*; %	*n*; %	
CC	3; 9.4%	28; 9.7%	*0.819*
GG	17; 53.1%	137; 47.4%	
CG	12; 37.5%	124; 42.9%	
**Allele**	Frequency	Frequency	
C	0.28	0.31	*0.620*
G	0.72	0.69	

*PTX3* (+ 281A > G)	Control	ESRD	*p* (*χ*^2^)
Genotype	*n*; %	*n*; %	
AA	5; 15.6%	72; 24.9%	*0.444*
GG	10; 31.3%	91; 31.5%	
AG	17; 53.1%	126; 43.6%	
**Allele**	Frequency	Frequency	
A	0.42	0.46	*0.491*
G	0.58	0.54	

*χ*^2^ Pearson’s Chi-square test, *ESRD* end-stage renal disease, *IL6* interleukin 6, *PTX3* pentraxin 3. Italic denotes *p-*values.

For *IL6* (-174G > C), the prevalence of CC, GG and CG genotypes, as well as the frequency of both alleles C and G was similar in ESRD patients and in healthy individuals. Likewise, regarding the *PTX3* (+ 281A > G) polymorphism, the genotype distribution of AA, GG and AG and allelic frequency of A and G did not differ between patients and controls. Additionally, in this cohort of renal patients, the genotype prevalence for both polymorphisms was not altered by gender, etiology of CKD, type of vascular access for dialysis procedure, dialysis vintage, dialysis type, diabetes, hypertension or CVD history (data not shown).

### Blood levels of the inflammatory markers and their association with the *IL6* (rs1800795) and *PTX3* (rs2305619) polymorphisms

The circulating levels of leukocytes, hsCRP, IL6, PTX3 and GDF15, observed for the different *IL6* and *PTX3* polymorphic genotypes, as well as gender, age and dialysis vintage, are presented in Table 4. For both polymorphisms, the patients showed no statistical differences in gender distribution, age or dialysis vintage among the different genotypes.

**Table 4:** Gender, age, blood levels of leukocytes, IL6, hsCRP, PTX3 and GDF15, according to the *IL6* and *PTX3* polymorphic genotype in end-stage renal disease patients (*n* = 289).

	*IL6* (-174G > C) genotype			*p* value		
	CC (*n* = 28)	GG (*n* = 137)	CG (*n* = 124)	CC *vs.* GG	CC *vs.* CG	GG *vs.* CG
**Gender, *n* (%)**						
Male	19 (67.9)	73 (53.3)	66 (53.2)	*0.337*		
Female	9 (32.1)	64 (46.7)	58 (46.8)			
**Age,** **years**	69.1 ± 13.7	68.7 ± 13.6	68.7 ± 13.7	*1.000*	*1.000*	*1.000*
**Dialysis** **vintage,** **years**	3.42 (1.59–8.97)	4.10 (1.64–7.45)	3.54 (1.67–6.71)	*0.561*	*0.436*	*0.593*
**Inflammatory biomarkers**						
Leukocytes × 10^9^/L	5.40 (4.42–6.60)	6.60 (5.46–7.92)	6.32 (5.32–7.68)	***0.006***	***0.017***	*0.417*
hsCRP (mg/dL)	0.52 (0.29–1.09)	0.42 (0.16–0.82)	0.30 (0.13–0.74)	*0.157*	***0.017***	*0.127*
IL6 (pg/mL)	4.23 (3.47–10.4)	4.45 (2.98–7.36)	3.68 (2.58–6.86)	*0.433*	*0.095*	*0.144*
PTX3 (ng/mL)	1.56 (1.08–2.43)	1.47 (0.96–2.08)	1.32 (0.99–1.86)	*0.309*	*0.176*	*0.533*
GDF15 (ng/mL)	13.6 (11.1–18.3)	10.2 (7.56–13.7)	10.5 (8.10–13.0)	** < *0.001***	** < *0.001***	*0.790*

	*PTX3* (+ 281A > G) genotype			*p* value		
	AA (*n* = 72)	GG (*n* = 91)	AG (*n* = 126)	AA vs. GG	AA vs. AG	GG vs. AG
**Gender, *n* (%)**						
Male	44 (61.1)	49 (53.8)	65 (51.6)	*0.425*		
Female	28 (38.9)	42 (46.2)	61 (48.4)			
**Age, years**	69.0 ± 14.2	70.5 ± 14.0	67.3 ± 12.9	*1.000*	*1.000*	*0.272*
**Dialysis vintage, years**	3.62 (1.83–6.76)	4.14 (2.01–7.47)	3.23 (1.46–7.52)	*0.469*	*0.501*	*0.167*
**Inflammatory biomarkers**						
Leukocytes × 10^9^/L	6.30 (5.38–7.68)	5.80 (5.10–7.64)	6.51 (5.40–7.60)	*0.437*	*0.603*	*0.189*
hsCRP (mg/dL)	0.50 (0.24–1.25)	0.28 (0.13–0.63)	0.38 (0.15–0.80)	** < *0.001***	***0.021***	*0.152*
IL6 (pg/mL)	4.76 (3.30–8.66)	3.62 (2.61–6.94)	4.03 (2.64–6.70)	***0.040***	*0.113*	*0.549*
PTX3 (ng/mL)	1.28 (1.02–1.86)	1.41 (0.85–1.86)	1.47 (0.98–2.26)	*0.987*	*0.430*	*0.424*
GDF15 (ng/mL)	10.4 (7.98–13.3)	11.4 (8.84–14.4)	10.2 (7.67–13.5)	*0.146*	*0.767*	*0.081*

Data are presented as mean ± standard deviation or as median (interquartile range). Multiple comparisons between genotypes were performed by the Mann–Whitney U test, by the one-way Anova with Bonferroni post-hoc tests or by the Pearson’s Chi-square test, as appropriate. Italic denotes *p*-values and bold-italic statistically significant *p-*values.

*GDF15* growth differentiation factor 15, *hsCRP* high-sensitivity C-reactive protein, *IL6* interleukin 6, *PTX3* pentraxin 3.

For the *IL6* (-174G > C) polymorphism, the highest levels of hsCRP, PTX3 and GDF15 were observed for the CC genotype; however, we only found significantly higher values of hsCRP in CC *versus* CG genotype, and significantly higher values of GDF15 in CC *versus* GG, and CC *versus* CG genotypes. Leukocytes were significantly lower in the CC carriers than in the other two genotypes.

Regarding the *PTX3* polymorphism, the AA genotype presented the highest values of hsCRP (significant for AA *versus* GG and AA *versus* AG) and IL6 (significant for AA *versus* GG), while GG genotype presented the lowest values of both biomarkers. There were no significant differences in the levels of leukocytes, GDF15 and PTX3 between genotypes for this SNP.

We also evaluated the correlation between the different inflammatory markers on study, within each polymorphic genotype (Table 5). In all *IL6* (-174G > C) genotypes, hsCRP was positively and significantly correlated with IL6; hsCRP also showed significant positive correlations with PTX3 and GDF15, but only for the GG genotype. The correlations between IL6 and PTX3, were positive for all genotypes, achieving significance only for GG genotype; considering IL6 and GDF15, the correlation was negative for CC genotype and positive for the other genotypes, reaching statistical significance only in CG genotype.

**Table 5:** Correlations between the inflammatory markers, within each *IL6* and *PTX3* polymorphic genotype, in end-stage renal disease patients (*n* = 289).

	Biomarker vs	IL6 (pg/mL)	PTX3 (ng/mL)	GDF15 (ng/mL)
***IL6* (-174G > C) genotype**				
CC (*n* = 28)	hsCRP (mg/dL)	0.472*	0.079	-0.101
GG (*n* = 137)		0.525***	0.187*	0.187*
CG (*n* = 124)		0.597***	0.047	0.165
CC (*n* = 28)	IL6 (pg/mL)	–	0.195	-0.203
GG (*n* = 137)		–	0.225**	0.132
CG (*n* = 124)		–	0.082	0.250**
***PTX3* (+ 281A > G) genotype**				
AA (*n* = 72)	hsCRP (mg/dL)	0.582***	0.038	-0.217
GG (*n* = 91)		0.576***	0.154	0.021
AG (*n* = 126)		0.546***	0.166	0.299**
AA (*n* = 72)	IL6 (pg/mL)	–	0.199	0.074
GG (*n* = 91)		–	0.118	-0.203
AG (*n* = 126)		–	0.066	0.274**

*p < 0.05; **p < 0.01; ***p < 0.001 correlations between parameters for each *IL6* or *PTX3* polymorphic genotypes (Spearman’s rank correlation *r*).

*GDF15* growth differentiation factor 15, *hsCRP* high-sensitivity C-reactive protein, *IL6* interleukin 6, *PTX3* pentraxin 3.

Concerning the *PTX3* (+ 281A > G) polymorphism (Table 5), hsCRP and IL6 were positively and significantly correlated for all genotypes; no significant correlations were found between hsCRP and PTX3 for any genotype; hsCRP and GDF15 showed a negative correlation for AA genotype, while in both GG and AG alleles presented a positive correlation that achieved a statistical significance in the AG genotype. For all genotypes, no statistically significant associations were observed between IL6 and PTX3; a significant positive correlation was observed between IL6 and GDF15 for GG carriers, and in the AG carriers we found a negative correlation, although without significance.

### *IL6* and *PTX3* genotype frequencies in deceased and alive ESRD patients (1-year follow-up)

During the one-year follow-up of the ESRD patients, 21 of them left the study (Table 1). In the remaining ESRD patients (*n* = 268), a total of 28 (10.4%) died due to several causes, including CVD, cachexia, infectious diseases, and other. The distributions of *IL6* and *PTX3* polymorphic genotypes in deceased and alive ESRD patients are shown in Table 6.

**Table 6:** Genotype frequencies in deceased and alive end-stage renal disease patients (1-year follow-up), according to *IL6* (rs1800795) and *PTX3* (rs2305619) polymorphisms.

	Deceased/alive [*n* (%)]	*p* (*χ*^2^)
***IL6* (-174G > C) genotype**		
CC	6/21 (22.2/77.8)	0.089
GG	13/113 (10.3/89.7)	
CG	9/106 (7.8/92.2)	
***PTX3* (+ 281A > G) genotype**		
AA	7/61 (10.3/89.7)	0.666
GG	7/78 (8.2/91.8)	
AG	14/101 (12.2/87.8)	

*χ*^2^ Pearson’s Chi-squared test, *IL6* interleukin 6, *PTX3* pentraxin 3.

**Figure 1:**
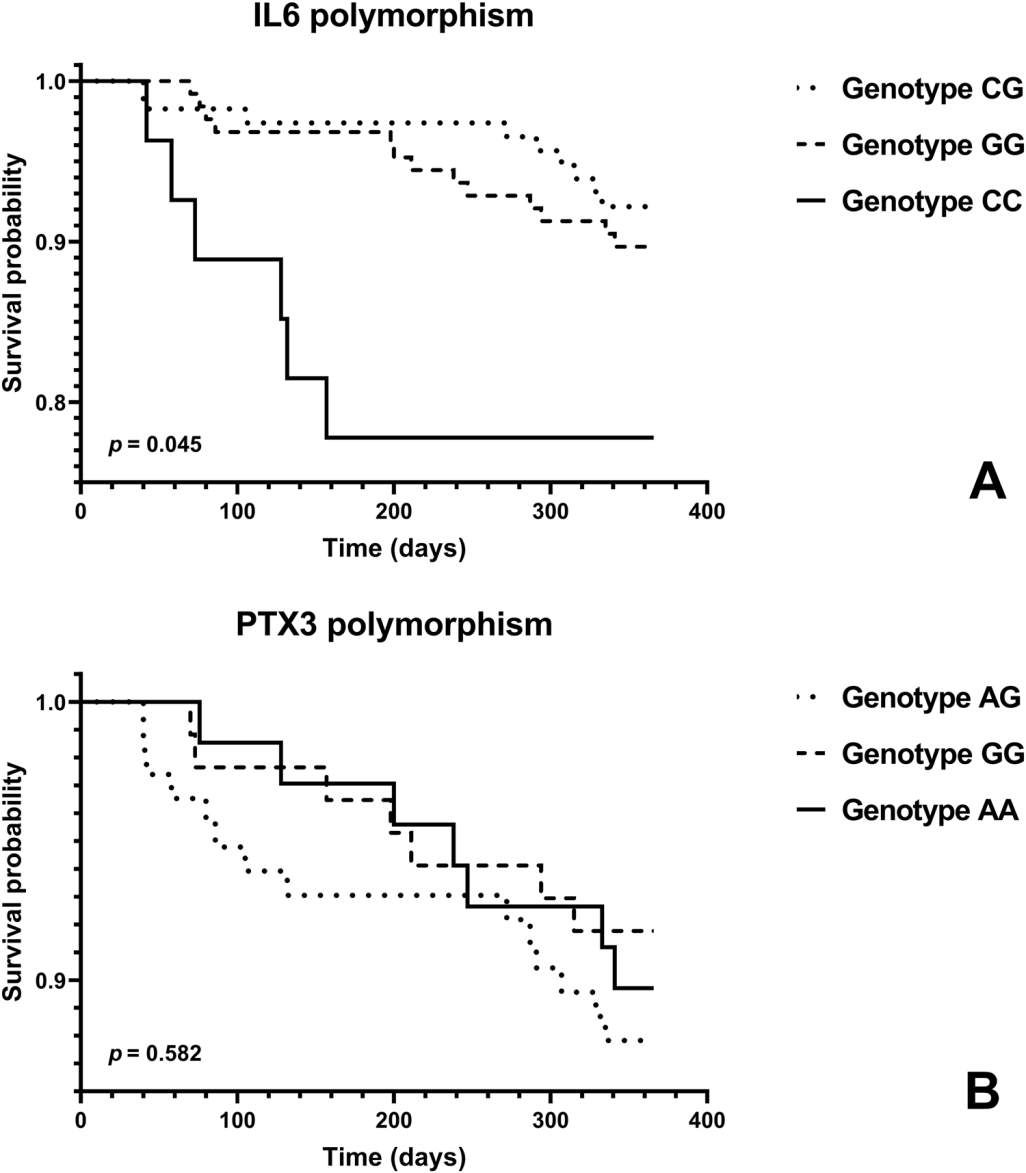
Survival cumulative curves for all-cause mortality in end-stage renal disease patients, by *IL6* **(A)** and by *PTX3* polymorphic genotype **(B)**. Survival distribution comparisons between genotypes was performed by the log-rank test.

Regarding the *IL6* rs1800795 SNP, the homozygous CC individuals showed the highest mortality rate (22.2%), followed by GG individuals (10.3%); while the heterozygous individuals showed the best outcome regarding mortality (7.8%). For individuals with the CC, GG, and CG genotype, the median survival time was 100 [54–138], 211 [83–290] and 291 [72–322] days, respectively(*p* = 0.023 for CC *versus* GG, *p* = 0.157 for CC *versus* CG, and *p* = 0.570 for CG *versus* GG). The survival cumulative curves for this polymorphism were statistically different (Fig. 1A).

Considering the *PTX3* polymorphism, the frequencies of AA, GG and AG genotypes were similar in both alive and deceased patients and there were no significant differences in mortality rates and in the survival cumulative curves between the different genotypes (Table 6 and Fig. 1B). The median survival time for individuals with AA, GG and AG genotypes was, respectively, 238 [129–333], 198 [73–294] and 118 [54–295] days (*p* = 0.338 for AA *versus* GG, *p* = 0.332 for AA *versus* AG and *p* = 0.654 for GG *versus* AG).

### Circulating levels of inflammatory markers according to *IL6* and *PTX3* polymorphic genotypes and mortality outcome

The blood levels of hsCRP, IL6, PTX3 and GDF15 in deceased and alive patients, according to the *IL6* and *PTX3* polymorphisms, are presented in Table 7. Considering the *IL6* SNP, when compared to alive patients, deceased patients with CC genotype showed significantly higher hsCRP concentration, while IL6 was significantly higher in GG and CG genotypes and PTX3 was significantly increased in the GG genotype. Regarding the *PTX3* polymorphism, for AA and AG genotypes, deceased patients presented significantly higher IL6 than alive patients and for GG and AG genotype patients PTX3 was significantly increased in deceased patients. No significant differences were observed for GDF15 levels for any *IL6* or *PTX3* genotypes between alive and deceased patients.

**Table 7:** Blood levels of IL6, hsCRP, PTX3 and GDF15 according to the *IL6* (rs1800795) and *PTX3* (rs2305619) single nucleotide polymorphisms in alive and deceased end-stage renal disease patients.

*IL6* (-174G > C) polymorphism	CC genotype (*n* = 27)		GG genotype (*n* = 126)		CG genotype (*n* = 115)	
1-year outcome	Alive (*n* = 21)	Deceased (*n* = 6)	Alive (*n* = 113)	Deceased (*n* = 13)	Alive (*n* = 106)	Deceased (*n* = 9)
hsCRP (mg/dL)	0.39 (0.29–0.80)	1.28* (0.55–3.64)	0.45 (0.16–0.82)	0.37 (0.29–1.10)	0.25 (0.13–0.68)	0.63 (0.14–1.64)
IL6 (pg/mL)	4.01 (3.06–10.9)	6.26 (3.62–22.4)	3.90 (2.78–7.06)	8.84** (4.48–15.0)	3.43 (2.54–6.72)	5.73* (3.77–11.6)
PTX3 (ng/mL)	1.41 (1.04–2.26)	2.18 (1.53–5.72)	1.28 (0.92–1.90)	1.96* (1.61–2.20)	1.33 (1.02–1.84)	2.13 (0.84–3.18)
GDF15 (ng/mL)	14.0 (12.0–18.2)	11.6 (8.19–34.6)	10.3 (7.66–13.7)	10.2 (7.26–13.3)	10.5 (8.28–13.1)	11.4 (8.56–13.2)

*PTX3* (+ 281A > G) polymorphism	AA genotype (*n* = 68)		GG genotype (*n* = 85)		AG genotype (*n* = 115)	
1-year outcome	Alive (*n* = 61)	Deceased (*n* = 7)	Alive (*n* = 78)	Deceased (*n* = 7)	Alive (*n* = 101)	Deceased (*n* = 14)
hsCRP (mg/dL)	0.50 (0.22–1.16)	0.98 (0.33–3.98)	0.27 (0.13–0.60)	0.50 (0.28–1.14)	0.35 (0.13–0.76)	0.70 (0.15–1.17)
IL6 (pg/mL)	4.57 (3.26–7.50)	14.3*** (11.3–22.0)	3.61 (2.53–7.02)	4.45 (3.87–8.05)	3.48 (2.58–6.18)	5.60* (3.74–8.87)
PTX3 (ng/mL)	1.26 (1.00–1.85)	1.71 (1.31–2.18)	1.32 (0.92–1.80)	2.23* (1.73–7.18)	1.41 (0.97–2.24)	2.06* (1.50–2.86)
GDF15 (ng/mL)	10.8 (7.92–13.5)	11.7 (8.50–14.2)	10.5 (8.28–13.1)	10.2 (4.89–14.5)	10.1 (7.64–13.6)	11.0 (8.64–13.6)

Data are presented as median (interquartile range). Multiple comparisons between genotypes were performed by the Mann–Whitney U test.

*p < 0.050. **p < 0.01 alive *versus* deceased.

*GDF15* growth differentiation factor 15, *hsCRP* high-sensitivity C-reactive protein, *IL6* interleukin 6, *PTX3* pentraxin 3.

The Cox regression survival analysis for all-cause mortality in ESRD (*n* = 268), using as reference the heterozygous patients for *IL6* polymorphism (Table 8), in an unadjusted model, showed that CC patients presented a significantly higher mortality risk with a HR of 3.275 [1.165 to 9.204]. In the model adjusted for age, dialysis vintage and type of vascular access, the CC genotype continued to show a significantly higher risk for mortality than the heterozygotes, with a HR of 2.961; the risk increased (HR = 3.356) when adjusting the model for the comorbidities, diabetes mellitus and history of CVD, besides all the previous confounding factors. The Cox regression analysis was not performed for *PTX3* polymorphism since we have already shown that no differences existed between genotypes in terms of mortality rate (Table 6) or survival cumulative curves (Fig. 1B).

**Table 8:** Cox regression analysis according to the *IL6* (rs1800795) polymorphism for all-cause mortality in end-stage renal disease patients (*n* = 268).

Cox regression^a^	Unadjusted model			Adjusted model^b^			Adjusted model^c^		
*IL6* (-174G > C) genotype	*p*	HR	95.0% CI for HR	*p*	HR	95.0% CI for HR	*p*	HR	95.0% CI for HR
CC	***0.024***	3.275	1.165–9.204	***0.042***	2.961	1.040–8.429	***0.022***	3.356	1.188–9.484
GG	*0.490*	1.349	0.577–3.157	*0.498*	1.342	0.573–3.140	*0.498*	1.342	0.573–3.145
CG	*0.072*	–	–	*0.119*	–	–	*0.065*	–	–

^a^Cox regression analysis using as reference the heterozygous genotype patients.

^b^Adjusted for age, dialysis vintage and vascular access.

^c^Adjusted for age, dialysis vintage, vascular access, and the co-morbidities, diabetes mellitus and history of cardiovascular disease.

*CI* confidence interval, *HR* hazard ratio, *IL6* interleukin 6.  Italic denotes *p*-values and bold-italic statistically significant *p*-values.

## Discussion

The identification and analysis of polymorphisms in genes that encode biochemical markers that are altered in CKD patients is important, since they might affect the outcome of the patients^1,12,13^. The inflammatory biomarkers IL6 and PTX3 are particularly enhanced in ESRD patients and have been associated with a higher risk for CVD events, the main cause of death in these patients; still, how the polymorphisms of their encoding genes affect the inflammatory response and patient’s outcome, remains poorly clarified. It is important to identify and validate common genetic variants and genotypes, to know if and how they influence patients’ outcome; this knowledge may provide more adequate interventions in patients at higher risk.

This study analyzed the allelic frequencies of two polymorphisms, namely *IL6* (-174G > C) and *PTX3* (+ 281A > G), in ESRD patients and in controls; evaluated their association with the degree of inflammation, shown by the blood levels of IL6, PTX3, CRP and GDF15, and with mortality risk, by recording events of death along one year.

For *IL6* rs1800795, the frequency of CC, GG and CG genotypes for patients and controls was similar (9.7%, 47.4% and 42.9%, in ESRD patients; 9.4%, 53.1% and 37.5%, in controls) (Table 3). A study in a healthy Polish population, reported that the frequencies of *IL6* (− 174 G > C) genotypes were 21.9% for CC, 28.8% for GG and 49.3% for CG, which were similar to those described for German and British populations; however the frequencies reported for Italian and American Caucasians, American Blacks and Asian Americans are similar to those observed in our study^32^, in which GG is the most common genotype and CC genotype the less frequent^33^. We found an allelic frequency of 0.28 and 0.31 for allele C and 0.72 and 0.69 for allele G, in controls and ESRD patients, respectively. According to the Reference SNP (rs) Report database for rs1800795, assessed on February, 25^th^ 2021 (www.ncbi.nlm.nih.gov/snp/rs1800795), these frequencies are 0.44/0.56 for alleles C/G in the European population; the allelic frequencies in our studied population are closer to those reported for southern European^34^ and North-American populations^35^.

For *PTX3* rs2305619, the genotype distribution was also similar between patients and controls (24.9%, 31.5% and 43.6% for AA, GG and AG, respectively, in ESRD patients; 15.6%, 31.3% and 53.2%, respectively, for controls). A study in healthy European subjects, reported that the distribution of AA, GG and AG genotypes was, respectively, 22.94%, 27.12% and 49.94%^25^. Another study involving Taiwanese controls showed 12.2%, 41.5% and 46.3%, for AA, GG and AG genotypes, respectively^29^. Our controls and ESRD patients presented similar genotype frequencies to those reported by Barbati et al*.*^25^. We also found that the allelic frequencies were 0.42 and 0.46 for allele A, and 0.58 and 0.54 for allele G, in controls and ESRD patients, respectively, which is in accordance with data obtained from Reference SNP (rs) Report database for rs2305619, assessed on February, 25^th^ 2021 (www.ncbi.nlm.nih.gov/snp/rs2305619), for a European population (allele A: 0.48 and allele G: 0.52). Thus, the allelic frequencies for *IL6* and *PTX3* polymorphisms in our patient cohort were similar to those described in other European populations. Moreover, as the genotype distribution between controls and ESRD patients (Table 3) was similar, it seems that none of the different alleles were more prevalent in ESRD patients. Additionally, both *IL6* and *PTX3* polymorphic genotype frequencies did not differ in ESRD patients, when sub-analyzed according to gender, etiology of CKD, type of vascular access for dialysis procedure, dialysis vintage, dialysis type, diabetes, hypertension and CVD history.

In our cohort of ESRD patients, *IL6* (-174G > C) polymorphic genotypes showed that the GG and CC genotypes presented a trend to higher circulating levels of IL6, although without reaching statistical significance (Table 4). Actually, data on literature about the effect of *IL6* (-174G > C) polymorphic genotypes on IL6 circulating levels is still controversial. In a study with Indian ESRD patients with malnutrition inflammation complex syndrome, the C allele was associated with higher IL6 levels, and both CC and CG genotypes conferred a higher (about threefold) mortality risk than the GG genotype; moreover, the increased levels of IL6, when associated with higher TNF-α and low IL-10 levels, appeared to contribute for the activation of inflammatory pathways that lead to higher disease susceptibility, poorer nutritional status and lower survival rate^16^. Another study, in a Southern Italian CKD cohort, also showed that patients with the CC genotype presented higher circulating levels of IL6 than those with CG or GG genotypes. Furthermore, CKD patients with CC genotype and high levels of IL6 showed a higher incidence rate (87%) of cardiovascular events, when compared to those with the CG or GG genotypes^19^. In contrast, some studies in patients with other inflammatory clinical conditions, have suggested that the homozygous genotype for C allele confers protection, as it was associated with lower IL6 circulating levels^36–40^. In a study in Korean patients on HD, the C allele of the *IL6* (-174 G/C) was not detected, and this absence did not seem to interfere in IL6 circulating levels, in spite of the high circulating levels of IL6 (about three times higher than those found in our patients)^17^. Different results were obtained in a study of Caucasian and African American ESRD patients on long-term dialysis, which showed the association of GG and CG genotypes with higher levels of IL6, as compared with those with the CC genotype^33^.

Concerning the effect of *IL6* (-174G > C) polymorphic genotypes on the other studied inflammatory biomarkers, we found that ESRD patients with CC genotype showed higher CRP levels (median: 0.52 mg/L), when compared to CG and GG genotypes (median: 0.42 mg/L and 0.30 mg/L, respectively), reaching statistical significance for CC *versus* CG (Table 4). Higher CRP values were also reported in CKD patients with CC genotype, compared with CG and GG genotypes, in other studies^19^. Comparing PTX3 and GDF15 levels between *IL6* polymorphic genotypes, we found that the CC genotype presented the highest median levels for both markers, reaching the increase a statistical significance for GDF15. Thus, our data suggest that the CC genotype for *IL6* (-174G > C) polymorphism contributes to an increased inflammatory response, by increasing CRP and GDF15 circulating levels in ESRD on dialysis. The circulating leukocytes are within normal reference values, presenting the CC genotype patients the lowest levels, further suggesting an altered inflammatory response for this genotype.

Higher GDF15 concentrations have been associated with mortality and with heart failure events^41^, as well as with CKD progression^42^. Also, there is evidence that CRP stimulates GDF15 expression in endothelial cells^43,44^. As already referred, our data show that, in ESRD patients, the CC genotype is associated to significantly increased GDF15 levels and a trend for higher CRP levels (Table 4); however, we found negative correlations between GDF15 and IL6/CRP for CC genotype patients, while for the other variants these correlations were positive (Table 5). This suggests that alternate transcription of *IL6* gene might lead to the activation of different metabolic pathways, which could have a direct impact on GDF15 circulating levels. We wonder about the importance of the influence of the *IL6* (-174G > C) polymorphism on GDF15 levels, which most certainly deserves further investigation.

The highest levels of hsCRP, PTX3 and GDF15 found in ESRD patients with the CC genotype, suggest that this genotype may increase the risk for CVD and for a poor outcome in ESRD patients. Epidemiologic studies have shown elevated levels of the inflammatory biomarkers, CRP, PTX3, and IL6 and GDF15^5,6,41^ in ESRD patients, and their association with the risk for CVD events and mortality^19,41,42,45–47^. Considering that the genotypic variations do not change over time, this highlights the importance of variants that although less frequent, may have biological value^46^.

The evaluation of PTX3 levels has been proposed as an important sensitive tool to predict cardiovascular mortality risk in patients with advanced CKD and to identify and treat early-stage subclinical atherosclerosis in these patients^5,24,48–50^. The PTX3 levels rise before traditional systemic inflammatory biomarkers, such as CRP, commonly used in laboratorial and clinical practice^6,51^. In our ESRD patient cohort, for the *PTX3* polymorphism, no statistically significant differences were found between PTX3 levels for the different genotypes. However, AA genotype presented the highest values of hsCRP and IL6, and GG genotype the lowest values (Table 4). As far as we know, no studies about the effect of this polymorphism have been performed in ESRD patients. Studies in patients at risk for myocardial infarction reported that this PTX3 polymorphism is associated with higher plasma PTX3 levels in individuals with AA genotype of rs2305619^25^. Actually, several studies have proposed PTX3 as an inflammatory biomarker associated with inflammation and CVD, and as an early marker of CV mortality in ESRD patients^6,51^*.* Moreover, elevated PTX3 levels have been also associated with lower eGFR and appear to independently predict incident CKD in the elderly, and, thus, appears to be a promising biomarker of kidney disease^49^. Our data show that the AA genotype, not only presents the highest levels of hsCRP and IL6, but also presents a positive significant correlation between them (Table 5), suggesting a higher risk for inflammation and possibly a poorer outcome.

As referred, along 1-year period we monitored patients, registering the events of death, in order to evaluate how the *IL6* and *PTX3* polymorphisms could affect their outcome. Along the one-year follow-up, 21 patients dropped from the study (e.g., transplant, recovering, abandoning KRT) and 28 patients died, showing an overall mortality rate of 10.4% (28 out of 268).

Evaluating mortality according to the *PTX3* (+ 281A > G) polymorphism, we found that the genotype frequency between deceased and alive patients did not differ and the mortality rates were similar between genotypes (AA: 10.3%; GG: 8.2%; AG: 12.2%) (Table 6). Considering the *IL6* (-174G > C) polymorphism, the genotype distribution in deceased patients was slightly different from that observed for survivors, almost reaching statistical significance (*p* = 0.089); the mortality rates per genotype were 22.2% for CC genotype, 10.3% for GG and 7.8% for CG (Table 6). The survival cumulative curves (Fig. 1A) are also strongly suggestive of a poorer outcome for patients with the CC genotype (*p* = 0.045) since the mortality rate in CC genotype carriers was more than twofold of that observed in the other two genotypes, which presented similar mortality rates, although a trend towards a lower value was observed in heterozygous individuals.

When comparing the circulating levels of the inflammatory markers in deceased and alive patients, we found that deceased patients with the *IL6* (-174G > C) CC genotype presented increased hsCRP (more than threefold). In the other two genotypes, the values of hsCRP were lower and similar to those presented by alive patients, but IL6 levels were higher. Thus, our data suggest an inappropriate inflammatory response to IL6-stimulus in CC genotype individuals, which might be associated to a less favorable outcome for their carriers (Table 7 and Fig. 1A). The cross-survival analysis (Cox regression) actually suggests that the IL6 polymorphic CC genotype is associated with a higher mortality risk, and heterozygosity with the lowest mortality risk (Table 8 and Fig. 1A). Performing Cox regression analysis and adjusting for confounding factors, such as age, dialysis vintage, type of vascular access, diabetes and history of CVD, the CC genotype remained as an independent predictor of death (Table 8, adjusted models). Our data suggest that ESRD patients with CC genotype have almost threefold higher risk for a poorer outcome, than the heterozygous. In line with our findings, a meta-analysis of 74 studies with 86,229 subjects, evaluated the association of -174G > C *IL6* (rs1800795) with the risk for CVD, and found that the C allele was associated with a higher risk for CVD^5,20^.

Comparing the circulating levels of the inflammatory markers in deceased and alive patients, according to the *PTX3* polymorphic genotypes, we found that deceased patients with the AA genotype presented the highest increase in IL6 (almost threefold than the survivors), the deceased GG genotype individuals showed increased PTX3 and the AG genotype deceased patients had higher IL6 and PTX3 levels; however, the mortality rates and survival cumulative curves for *PTX3* polymorphism, were similar in all genotypes (Table 6 and Fig. 1B). Thus, in spite of the enhancement in the inflammatory markers, especially the striking increase in IL6 for AA genotype, in deceased patients, we did not find a significant impact of the *PTX3* polymorphism in the outcome of these patients.

This study comprises some limitations that should be considered when evaluating the relevance of the presented data. The number of healthy subjects included in the Control group is limited and, notwithstanding the reasonable number of patients included in the study, the sample size of this cohort is also reduced, particularly when subdividing samples for data analysis by genotype. The short follow-up period (1 year) further limits the sample size for outcome (all-cause mortality) analysis. Still, our patients’ cohort is representative of the Portuguese ESRD population, and the range of values observed for each circulating inflammatory parameters in both groups are consistent with those of other studies, thus supporting the validity of the present findings. Furthermore, although gene sequencing is considered the gold standard diagnostic method for identification of germline mutations in genes with high allelic heterogeneity, real-time PCR TaqMan SNP genotyping assays were used in this study. Nevertheless, this technique has been proven to be cost-effective and less time-consuming, while being reliable and accurately discriminating alleles using small amounts of DNA.

## Conclusions

Our data show that both *IL6* (-174G > C) (r1800795) and *PTX3* (+ 281A > G) (rs2305619) polymorphisms seem to modulate the inflammatory response in ESRD patients. The CC genotype, the less frequent genotype for *IL6* polymorphism, appears to enhance the inflammatory response in ESRD patients, and is associated with a less favorable outcome. Our study suggests that inflammation can be induced by underlying individual genetic characteristics and highlights the importance of research on variants that, although less frequent, may have biological value, as it appears to be the case of CC genotype for *IL6* polymorphism.

## References

[1] Corredor Z (2020). Genetic variants associated with chronic kidney disease in a Spanish population. Sci. Rep..

[2] Levey AS (2011). The definition, classification, and prognosis of chronic kidney disease: A KDIGO controversies conference report. Kidney Int..

[3] Welfare, A. I. o. H. a. Projections of the prevalence of treated end-stage kidney disease in Australia, 2012–2020. In *Cat.**No.**PHE**176.* 1–51 (AIHW, 2014).

[4] Liyanage T (2015). Worldwide access to treatment for end-stage kidney disease: A systematic review. Lancet.

[5] Dai L, Golembiewska E, Lindholm B, Stenvinkel P (2017). End-stage renal disease, inflammation and cardiovascular outcomes. Contrib. Nephrol..

[6] Valente MJ (2019). Long Pentraxin 3 as a broader biomarker for multiple risk factors in end-stage renal disease: Association with all-cause mortality. Mediators Inflamm..

[7] Ho JE (2013). Biomarkers of cardiovascular stress and incident chronic kidney disease. Clin. Chem..

[8] Su, H., Lei, C. T. & Zhang, C. Interleukin-6 signaling pathway and its role in kidney disease: An update. *Front.**Immunol.* **8**. 10.3389/fimmu.2017.00405 (2017). 10.3389/fimmu.2017.00405 PMC5399081 28484449

[9] Kaminska J (2019). IL 6 but not TNF is linked to coronary artery calcification in patients with chronic kidney disease. Cytokine.

[10] Lousa, I. *et**al.* New potential biomarkers for chronic kidney disease management-A review of the literature. *Int.**J.**Mol.**Sci.* **22**. 10.3390/ijms22010043 (2020). 10.3390/ijms22010043 PMC7793089 33375198

[11] Sun J (2016). Biomarkers of cardiovascular disease and mortality risk in patients with advanced CKD. Clin. J. Am. Soc. Nephrol..

[12] Kottgen A (2009). Multiple loci associated with indices of renal function and chronic kidney disease. Nat. Genet..

[13] Okada R (2012). Pro-/anti-inflammatory cytokine gene polymorphisms and chronic kidney disease: A cross-sectional study. BMC Nephrol..

[14] Rao M (2007). Cytokine gene polymorphism and progression of renal and cardiovascular diseases. Kidney Int..

[15] Al-Radeef MY, Allawi AAD, Fawzi HA (2018). Interleukin-6 gene polymorphisms and serum erythropoietin and hemoglobin in hemodialysis Iraqi patients. Saudi J. Kidney Dis. Transplant..

[16] Sharma R, Agrawal S, Saxena A, Sharma RK (2013). Association of IL-6, IL-10, and TNF-alpha gene polymorphism with malnutrition inflammation syndrome and survival among end stage renal disease patients. J. Interferon Cytokine Res..

[17] Ryu JH, Kim SJ (2012). Interleukin-6 -634 C/G and -174 G/C polymorphisms in Korean patients undergoing hemodialysis. Korean J. Intern. Med..

[18] Neelofar K, Ahmad J, Ahmad A, Alam K (2017). Study of IL4-590C/T and IL6-174G/C gene polymorphisms in type 2 diabetic patients with chronic kidney disease in North Indian population. J. Cell Biochem..

[19] Spoto B (2015). Association of IL-6 and a functional polymorphism in the IL-6 gene with cardiovascular events in patients with CKD. Clin. J. Am. Soc. Nephrol..

[20] Gonzalez-Castro TB (2019). Interleukin 6 (rs1800795) gene polymorphism is associated with cardiovascular diseases: A meta-analysis of 74 studies with 86,229 subjects. EXCLI J..

[21] Hassan MO (2019). Interleukin-6 gene polymorhisms and interleukin-6 levels are associated with atherosclerosis in CKD patients. Clin. Nephrol..

[22] Introna M (1996). Cloning of mouse ptx3, a new member of the pentraxin gene family expressed at extrahepatic sites. Blood.

[23] Bottazzi, B. *et**al.* Multimer formation and ligand recognition by the long pentraxin PTX3. Similarities and differences with the short pentraxins C-reactive protein and serum amyloid P component. *J.**Biol.**Chem.* **272**, 32817–32823. 10.1074/jbc.272.52.32817 (1997). 10.1074/jbc.272.52.32817 9407058

[24] Speeckaert MM, Speeckaert R, Carrero JJ, Vanholder R, Delanghe JR (2013). Biology of human pentraxin 3 (PTX3) in acute and chronic kidney disease. J. Clin. Immunol..

[25] Barbati E (2012). Influence of pentraxin 3 (PTX3) genetic variants on myocardial infarction risk and PTX3 plasma levels. PLoS ONE.

[26] Gu HF (2019). Genetic and epigenetic studies in diabetic kidney disease. Front. Genet..

[27] Zhu H (2017). Association of Pentraxin 3 gene polymorphisms with susceptibility to diabetic nephropathy. Med. Sci. Monit..

[28] Dharma S (2019). The 3q25 rs2305619 polymorphism is associated with coronary microvascular obstruction following primary angioplasty for acute ST-segment-elevation myocardial infarction. Circ. Cardiovasc. Interv..

[29] Yeh CM (2019). Functional genetic variant of long Pentraxin 3 gene is associated with clinical aspects of oral cancer in male patients. Front. Oncol..

[30] Badr EA, Hamoda GE, Tayel SI, Elshayeb EI (2017). Association of genetic variants of pentraxin 3 rs3816527 with hypertension in chronic kidney disease patients. Mol. Cell Biochem..

[31] Zandifar A, Iraji N, Taheriun M, Tajaddini M, Javanmard SH (2015). Association of the long pentraxin PTX3 gene polymorphism (rs3816527) with migraine in an Iranian population. J. Neurol. Sci..

[32] Kurzawski M (2005). Frequencies of the common promoter polymorphisms in cytokine genes in a Polish population. Int. J. Immunogenet..

[33] Balakrishnan VS (2004). Cytokine gene polymorphisms in hemodialysis patients: Association with comorbidity, functionality, and serum albumin. Kidney Int..

[34] Poli F (2002). Allele frequencies of polymorphisms of TNFA, IL-6, IL-10 and IFNG in an Italian Caucasian population. Eur. J. Immunogenet..

[35] Hoffmann SC (2002). Ethnicity greatly influences cytokine gene polymorphism distribution. Am. J. Transplant..

[36] Fishman D (1998). The effect of novel polymorphisms in the interleukin-6 (IL-6) gene on IL-6 transcription and plasma IL-6 levels, and an association with systemic-onset juvenile chronic arthritis. J. Clin. Invest..

[37] Hulkkonen J, Pertovaara M, Antonen J, Pasternack A, Hurme M (2001). Elevated interleukin-6 plasma levels are regulated by the promoter region polymorphism of the IL6 gene in primary Sjogren's syndrome and correlate with the clinical manifestations of the disease. Rheumatology (Oxford).

[38] Libra M (2006). Analysis of G(-174)C IL-6 polymorphism and plasma concentrations of inflammatory markers in patients with type 2 diabetes and peripheral arterial disease. J. Clin. Pathol..

[39] Lim CS (2002). The -174 G to C polymorphism of interleukin-6 gene is very rare in Koreans. Cytokine.

[40] Pramudji H, Demes CM, Dewi K, Tasmini T, Ahmad HS (2019). Association of -174 G>C interleukin-6 gene polymorphism with interleukin-6 and c-reactive protein levels and obesity: A case-control study among people/residents of Western Indonesia. Med. J. Malaysia.

[41] Tuegel C (2018). GDF-15, Galectin 3, soluble ST2, and risk of mortality and cardiovascular events in CKD. Am. J. Kidney Dis..

[42] Nair V (2017). Growth differentiation factor-15 and risk of CKD progression. J. Am. Soc. Nephrol..

[43] Kim JS, Kim S, Won CW, Jeong KH (2019). Association between plasma levels of growth differentiation factor-15 and renal function in the elderly: Korean Frailty and Aging Cohort Study. Kidney Blood Press. Res..

[44] Kim Y, Noren Hooten N, Evans MK (2018). CRP stimulates GDF15 expression in endothelial cells through p53. Mediators Inflamm..

[45] Benes J (2019). The role of GDF-15 in heart failure patients with chronic kidney disease. Can. J. Cardiol..

[46] Liu Y (2006). IL-6 haplotypes, inflammation, and risk for cardiovascular disease in a multiethnic dialysis cohort. J. Am. Soc. Nephrol..

[47] Wang K (2020). Cardiac biomarkers and risk of mortality in CKD (the CRIC study). Kidney Int. Rep..

[48] Tong M (2007). Plasma pentraxin 3 in patients with chronic kidney disease: associations with renal function, protein-energy wasting, cardiovascular disease, and mortality. Clin. J. Am. Soc. Nephrol..

[49] Sjoberg B (2016). Association between levels of pentraxin 3 and incidence of chronic kidney disease in the elderly. J. Intern. Med..

[50] Casula M (2017). Update on the role of Pentraxin 3 in atherosclerosis and cardiovascular diseases. Vascul. Pharmacol..

[51] Krzanowski M (2017). Pentraxin 3 as a new indicator of cardiovascular related death in patients with advanced chronic kidney disease. Pol. Arch. Intern. Med..

